# Early life metal exposure dysregulates cellular bioenergetics in children with regressive autism spectrum disorder

**DOI:** 10.1038/s41398-020-00905-3

**Published:** 2020-07-07

**Authors:** Richard E. Frye, Janet Cakir, Shannon Rose, Leanna Delhey, Sirish C. Bennuri, Marie Tippett, Raymond F. Palmer, Christine Austin, Paul Curtin, Manish Arora

**Affiliations:** 1 Barrow Neurological Institute at Phoenix Children’s Hospital, Phoenix, AZ USA; 2 University of Arizona College of Medicine – Phoenix, Phoenix, AZ USA; 3 North Carolina State University, Raleigh, NC USA; 4 Arkansas Children’s Research Institute, Little Rock, AR USA; 5 Department of Pediatrics, University of Arkansas for Medical Sciences, Little Rock, AR USA; 6 Department of Family and Community Medicine, University of Texas Health Science Center, San Antonio, TX USA; 7 Department of Environmental Medicine and Public Health, Icahn School of Medicine at Mount Sinai, New York, NY USA

**Keywords:** Autism spectrum disorders, Physiology, Human behaviour

## Abstract

Neurodevelopmental regression (NDR) is a subtype of autism spectrum disorder (ASD) that manifests as loss of previously acquired developmental milestones. Early life dysregulation of nutritional metals and/or exposure to toxic metals have been associated with ASD, but the underlying biological mechanisms by which metals influence neurodevelopment remain unclear. We hypothesize that metals influences neurodevelopment through dysregulation of bioenergetics. Prenatal and early postnatal metal exposures were measured using validated tooth-matrix biomarkers in 27 ASD cases (13 with NDR) and 7 typically-developing (TD) controls. Mitochondrial respiration and glycolysis were measured in peripheral blood mononuclear cells using the Seahorse XF96. Children with ASD demonstrated lower prenatal and postnatal Copper (Cu) and prenatal Nickel concentrations and Copper-to-Zinc (Cu/Zn) ratio as compared with TD children. Children with ASD and NDR showed greater metal-related disruption of cellular bioenergetics than children with ASD without NDR. For children with ASD and NDR mitochondrial respiration decreased as prenatal Manganese concentration increased and increased as prenatal Zinc concentration increased; glycolysis decreased with increased exposure to prenatal Manganese and Lead and postnatal Manganese. For children with ASD without a history of NDR, glycolysis increased with increased postnatal exposure to Tin. Language and communication scores in children with ASD were positively related to prenatal Cu exposure and Cu/Zn ratio. This study suggests that prenatal nutritional metals may be important for neurodevelopment in children with ASD, and that exposure to toxic metals and differences in nutritional metal exposures is associated with dysregulation of cellular bioenergetics, particularly in the NDR subtype of ASD.

## Introduction

Autism spectrum disorder (ASD) is a behaviorally defined disorder, which is associated with significant pathophysiology^1^. Some studies estimate ASD to affect 1 in 45 individuals in the United States^2^. Although ASD research has focused on genetics^1^, recent estimates suggest that inherited single gene and chromosomal defects only account for a minority of ASD cases^3^ and that ASD most likely arises from a complicated interaction between genetic predisposition and the environment^4,5^. Although numerous environmental agents have been epidemiologically associated with ASD, the mechanism by which such agents cause neurodevelopmental disorders is understudied and unclear^6^.

Commonly studied environmental toxicants include gestation and early-life exposure to fine air particulate matter (e.g., PM_2.5_), which is linked to an increase in ASD risk^7–9^. Important nutrients linked to ASD include folate, which increases the risk for ASD when not provided in sufficient amounts during gestation^10^. Recent research has demonstrated that exposure to metals, which can be nutritional and/or toxic during gestation and early life, can increase the risk of developing ASD^11,12^. Lead (Pb) exposure is associated with neurodevelopmental disorders including behavioral problems, learning deficits, intellectual disability, and ASD^13^. Other metals such as Zinc (Zn), Copper (Cu), and Manganese (Mn) are essential for metabolic systems and neurodevelopment but can be toxic in excessive amounts^14^. A recent major advancement that has helped with the study of exposure to metals prenatally and during early life is the ability to directly measure exposure with a week-by-week temporal resolution during critical developmental periods by examining metal deposits in deciduous teeth^15^. Recent studies using deciduous teeth have confirmed that prenatal disruption in these metals are linked to an increase in the risk of developing ASD^11,12^.

Although toxic and nutritional metal exposure has been associated with an increased risk of neurodevelopmental and neuropsychiatric disorders, the exact pathophysiological mechanisms by which metal exposure contributes to these abnormalities are mostly based on animal models and not well understood in humans. One important mechanism by which environmental influences, especially those linked to ASD, can affect biological systems is through mitochondrial metabolism^6,16^. This is compelling since up to 30–50% of individuals with ASD demonstrate biomarkers of abnormal mitochondrial function^16,17^, and up to 80% of individuals with ASD show abnormal electron transport chain (ETC) activity in lymphocytes^18^, granulocytes^19^, and post-mortem brain^20^. Furthermore, the great majority of cases of individuals with ASD and mitochondrial disease ***do not*** have identified genetic mutations, suggesting that alternations in mitochondrial function may be driven from nongenetic influences such as the environment^16^. Interestingly, the same environmental agents that have been linked to ASD can also induce mitochondrial metabolism abnormalities^21^. Furthermore, mitochondria adapt to changes in the microenvironment through a process called mitoplasticity, which has the potential to result in long-term changes to mitochondrial function^22^.

In this study, we directly measure both toxic and nutrient metals deposited into each participant’s deciduous teeth during gestation and early in infancy (first 9 months of age) to determine if such metals have a long-term association with changes in bioenergetics in children with ASD. About one-third of children with ASD fall into the neurodevelopmental regression (NDR) subtype. Children with NDR demonstrate normal development in infancy but then lose social and language skills, usually between the first and second years of life. Because NDR is associated with mitochondrial dysfunction in children with^16,23^ and without^24^ ASD, we pay special attention to children with ASD diagnosed with NDR.

As the mitochondria is particularly sensitive to environmental factors such as nutrient and toxic metal exposure, and since mitochondrial dysfunction is linked to NDR, it is hypothesized that prenatal and early postnatal exposure to metals will result in long-term changes in mitochondrial function that is sustained into childhood, particularly in those children with ASD and NDR. In this study, we demonstrate that metal exposure during gestation is related to physiological changes in bioenergetics that persist into childhood for children with ASD, primarily in those with a history of NDR. These results suggest that early life environmental exposures are linked to long-term physiological abnormalities in a subset of children with ASD.

## Methods

### Participants

Protocols registered in clinicaltrials.gov as NCT02000284 and NCT02003170 and approved by the Institutional Review Board at the University of Arkansas for Medical Sciences (Little Rock, AR) were used in this study. Exclusion criteria were (i) chronic treatment with medications that would detrimentally affect mitochondrial function such as antipsychotic medications; (ii) vitamin or mineral supplementation exceeding the recommended daily allowance, and (iii) prematurity.

Inclusion criteria included: (a) a diagnosis of ASD and (b) <18 years of age. The ASD diagnosis was defined by one of the following: (i) a gold-standard diagnostic instrument: the Autism Diagnostic Observation Schedule or Autism Diagnostic Interview-Revised; (ii) the state of Arkansas diagnostic standard, defined as agreement of a physician, psychologist, and speech therapist with experience in diagnosing ASD; and/or (iii) Diagnostic Statistical Manual diagnosis by a physician along with standardized validated questionnaires and diagnosis confirmation by the first author (REF). We have previously validated that criteria (ii) and (iii) captures an accurate diagnosis of ASD by showing that participants defined using criteria (ii) or (iii) are well within the diagnostic criteria for ASD using the Autism Diagnostic Interview-Revised^25^.

Parents of participants provided written informed consent. Children underwent a fasting blood draw in the morning. Control individuals did not have any neurological disorders or developmental delays and none of the individuals included in this study were diagnosed with mitochondrial disease. Patients were recruited from a nationally recognized multispecialty ASD clinic. Patients lived in one of eight states (AL, AR, FL, GA, IL, KY, MO, and TX) or Canada. Supplementary Table S1 outlines the participant characteristics. Our recent publications outline the methodology for rating participant characteristics of this participant cohort^25–28^. The numerous medical conditions were summarized into categories. Feeding problems, diarrhea, constipation, stomach and/or abdominal pain, vomiting, and gastroesophageal reflux disease were considered gastrointestinal disorders. Asthma, allergies, allergic skin conditions, breathing problems, and other lung problems were considered allergic disorders. Depressive, anxiety, bipolar, obsessive compulsive, and attention deficit with or without hyperactivity disorders were considered psychiatric disorders. Recurrent sinusitis, ear infections, throat infections, and other immune problems were considered immune disorders. Headaches, hearing problems, microcephaly, vision problems, macrocephaly, strabismus, seizures, epilepsy, opthalmoplegia, tics, cerebral palsy, and muscle weakness were considered neurological disorders. Cardiovascular disorders are considered syncope, heart failure, and congenital heart disease. Growth disorders are considered failure to thrive, growth hormone deficiency, short and tall stature, overweight, and obesity. Endocrine disorders are considered thyroid or other endocrine problems.

The NDR history was obtained using the Developmental and Neurobehavioral Regression (DANR) questionnaire, which has been developed as part of our ASD research program. The DANR records detail information about NDR including specific questions on premorbid functioning before the regression, duration of the regression, specific skills lost and when the skills were regained, whether there was a single or multiple regressions and any known trigger such as illness, fever, or seizure. The two ASD groups were similar in characteristics except for gastrointestinal medications, which were used in a greater percentage of children with ASD and NDR as compared with those with ASD without NDR.

Contemporaneous controls were slightly younger and had more females than the children with ASD. The control children were generally healthy children without significant chronic health conditions although one had a history of strabismus, two had a history of headache, one had anxiety, and several had seasonal allergies. Most (5 of 7) of these individuals underwent the Vineland Adaptive Behavior Scale 2nd Edition to document their normal development. In addition, the controls were screened with the social communication questionnaire to document the lack of ASD symptoms with a total score <12.

Metal exposure was measured from a total of 34 children, including 7 who were typically developing (TD) [2 siblings of children with ASD and 5 unrelated to the children with ASD]. Neither sex nor age were significant covariates in any analysis. Mitochondrial respiration and glycolysis were measured in 27 children who were diagnosed with ASD without known mitochondrial disease. Some children provided repeated blood samples for analysis: 17 provided one sample, 9 provided two samples and 1 provided three or more samples. Participants were only required to provide one blood sample but could provide up to five repeated samples.

### Neurodevelopmental and behavioral measurements

Common validated measures were used to assess neurodevelopment and behavior in children with ASD^25,29^. Core language was assessed by the most appropriate instrument given the participants age, either the Clinical Evaluation of Language Fundamentals (CELF)-preschool-2, CELF-4, or the Preschool Language Scale-5 (PLS-5). The standardized summary score of each instrument (mean 100, standard deviation 15) was used as the measure of core language similar to our previous study^25^. The Vineland Adaptive Behavior Scale (VABS) 2nd edition Survey Interview Form was used as a measure of neurodevelopment. Parents completed the Aberrant Behavior Checklist (ABC) and the Social Responsiveness Scale (SRS) as measures of behavior and ASD symptoms. For language and VABS, higher scores represent higher functioning and for the SRS and ABC lower scores represent less impairment.

### Measurements of prenatal metal exposure

Elemental bio-imaging using laser ablation-inductively coupled plasma mass-spectrometry (LA-ICP-MS) measured concentrations of Pb (lead), Ni (nickel), Cr (chromium), Mg (magnesium), Mn (manganese), Cu (copper), Zn (zinc), Sr (strontium), Sn (tin), and Ba (barium) in deciduous teeth with a trimester-by-trimester resolution^30–33^. We also calculated the Cu-to-Zn ratio. Many studies in ASD have used the Zn-to-Cu ratio but we found in tooth data that the Zn-to-Cu ratio had very wide variability and a non-normal distribution. Thus, we used the Cu-to-Zn ratio which demonstrates a more normal distribution.

**Fig. 1:**
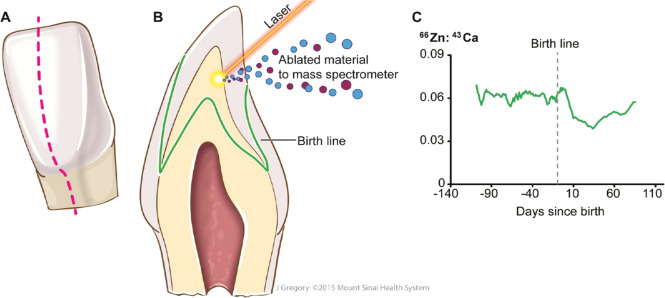
Overview of tooth-matrix biomarkers applied in this study. **a** Plane in which teeth were sectioned. **b** Laser ablation-inductively coupled plasma mass spectrometry analysis. **c** Output data where developmental timing is assigned to elemental profiles in each sample.

The LA-ICP-MS system includes an Agilent 8800 ICP-MS and a New Wave Nd:YAG 193 nm laser ablation system with a detection limit of 0.05 µg/g for the metals studied (Fig. 1). All samples analyzed include calibration standards and quality control (QC) samples. Certified reference materials (e.g., NIST 2974 freeze-dried tissue for persistent pollutants and NIST 3673 non-smokers’ urine for phthalates, phenols, and tobacco metabolites) are used for QC and method validation. Isotopically labeled internal standards monitor analyte recovery during sample preparation and are used to correct for matrix-effects during analysis. Either gas chromatography (GC)-MS/MS or liquid chromatography (LC)-MS/MS determined analytes, depending on sensitivity and lowest detection limit achievable. Traditionally, polar analytes are determined by LC, and non-polar and volatiles by GC. Tooth metal biomarkers, particularly the method used in this study, has been utilized in previous studies of prenatal metal exposure^11,12^. Higher values indicate higher metal concentrations in the tooth at a particular temporal window.

Tooth metal biomarkers have been validated using several methods. First, abrupt changes in Mn and Zn concentrations occur at birth; tooth-matrix biomarkers have demonstrated that this abrupt change in Mn and Zn concentrations in deciduous teeth is congruent with the neonatal line, a histological feature formed in enamel and dentine^11,34,35^. Second, monozygotic twin studies have verified the correspondence between fetal exposure and metal deposits in deciduous teeth^11^. Third, metal deposition in teeth has been compared with other environmental matrices in prospective pregnancy cohorts^15,32,34–36^. Fourth, blood lead levels measured from the mother prenatally and children postnatally correspond to deciduous tooth measurements^15^. Finally, animal studies with controlled exposures have been used to validate the method^35,37^.

### Blood collection and processing

Up to 20 ml of blood was collected into an ethylenediaminetetraacetic acid-Vacutainer tube, chilled on ice and centrifuged at 1500 g for 10 min at 4 °C to separate plasma within 15 min of collection. Plasma was removed and stored at −80 °C for later analysis. Plasma was replaced with room temperature wash buffer containing Ca^+2^/Mg^+2^-free PBS with 0.1% BSA and 2 mM ethylenediaminetetraacetic acid. Diluted blood was then layered on top of Histopaque-1077 (Sigma Aldrich, St. Louis, MO, USA) and centrifuged at 400 g for 30 min at room temperature. Peripheral blood mononuclear cells (PBMCs) were washed twice with wash buffer and counted using a hemocytometer.

### Bioenergetic assay

Bioenergetic data was obtained from PBMCs using a state-of-the-art Seahorse 96XF Analyzer (Seahorse Bioscience, Inc., North Billerica, MA) that measures oxygen consumption rate in real-time in a 96-well plate. This analyzer evaluates a wide range of intact living cell types^38,39^ and can measure ETC, glycolytic, and fatty-acid oxidation function^40^. The assay measures several key respiratory parameters: (a) Adenosine triphosphate-Linked Respiration, (b) Proton leak Respiration, (c) Maximal Respiratory Capacity, a parameter that is sensitive to deficits in mitochondrial biogenesis, mtDNA damage and/or inhibition of ETC function, and (d) Reserve Capacity, a parameter which determines the threshold at which bioenergetic dysfunction occurs. Measure of glycolysis are also measured. The extracellular acidification rate, a measure of lactate production, is equated to the glycolytic rate (i.e., glycolysis) and measured simultaneously with oxygen consumption rate in the Seahorse assay before the injection of oligomycin.

PBMCs were placed in assay media (unbuffered RPMI supplemented with 1 mM pyruvate, 2 mM glutamate, and 25 mM glucose) and plated at ~4 × 10^5^ cells per well of a poly-D-lysine coated XF96 well plate. For each experimental condition, four samples were measured simultaneously to improve assay reliability. Runs with clear measurement probe failure or reagent injection failure were eliminated. Overall, there was a total of 120 bioenergetic measurements used in the analysis.

### Statistical analysis

Analyses were performed using SAS 9.4 (SAS Institute Inc., Cary, NC) and PAWS Statistics 18 (SPSS Inc, Quarry Bay, HK). Graphs were produced using Excel version 16.0 (Microsoft Corp, Redmond, WA). In general, several personnel specializing in specific parts of data collection and analysis were used to maintain blinding to the outcome measures when possible. Specifically the blood was collected and relevant clinical information was obtained by different people (L.D., M.T.) from the laboratory personnel who performed the mitochondrial respiratory measurement (S.R., S.C.B.) and the laboratory that performed the tooth analysis (M.A.). Data from all sources was compiled by non-laboratory staff (L.D., M.T.) and statistical analysis was performed by individuals not directly involved in sample collection or laboratory analysis of the samples (J.C., R.E.F.).

A linear mixed-model was used to account for both within-subject variation from repeated mitochondrial measurements on the same individual (replicates) and the between-subject variation from variables such as NDR and metal concentration. Variances between groups were similar and data was normally distributed. All interactions were considered in the models and the final models were simplified to eliminate any non-significant higher order interactions. Because of the multiple metals analyzed the overall *p* was set to ≤0.01 for all analyses. To verify this approach, we calculated the False Discovery Rate for each set of statistical tests for each respiratory parameter on all metals. A False Discovery Rate of 5% is consistent with setting an *α* ≤ 0.01 significance level^41^.

Since multiple mitochondrial respiratory measurements were investigated, the metal investigated needed to demonstrate significance for at least three of the four respiratory measurements. This is a conservative approach to decreasing false positives. Since only one measure of glycolysis was investigated, this increase stringent criteria of having multiple respiratory parameters positive could not be set for the glycolysis analysis.

In the case of an interaction between NDR with metal concentration, the *p* value for the follow-up correlation between the respiratory parameter and the metal concentration for the group (NDR or not NDR) was required to be ≤0.01. To confirm and complement the mixed-model analysis, bivariate Pearson correlation coefficient *r* along with the *p* value for the *r* are presented in the scatterplot graphs. The *r* values can be used as a representation of effect size since *r* values are already standardized measures of the strength of the relationship^42^. Accordingly, *r* of 0.1–0.3 is recognized as a small effect, *r* of 0.3–0.5 is recognized as a medium effect and *r* of 0.50 or above is recognized as a large effect.

It is important to realize that the small sample size of this study limits the power of the statistical analyses. Thus, we cannot rule out relationships that are not found to be statistically significant. However, the purpose of this study is to determine if there are relationships that do exist given the conservative false positive rate (i.e., ≤1%).

As an exploratory analysis we have examined the relationship between neurodevelopmental and behavioral measures, and metals found to be important in ASD and/or mitochondrial function using linear regression including NDR as a moderating variable. For this exploratory analysis, alpha was set at 5%. Follow-up bivariate Pearson correlation were also computed to verify the analysis with relationships requiring an alpha of 5%.

## Results

We compared the concentration of metals in deciduous teeth between children with ASD and TD control children as well as between children with ASD with and without a history of NDR (See Supplementary Table S2). There were no differences in tooth metal concentrations in the prenatal or postnatal period between those with and without NDR. However, compared with TD children, ASD children demonstrated significantly lower Ni, Cu, and Cu-to-Zn ratio during the prenatal period and lower Cu during the early postnatal period. Zn and Sn were also lower in ASD children during the prenatal period, although the difference did not reach our stringent (*p* ≤ 0.01) criteria (See Supplementary Material for Statistics).

### Mitochondrial respiration and prenatal metal exposure

**Fig. 2:**
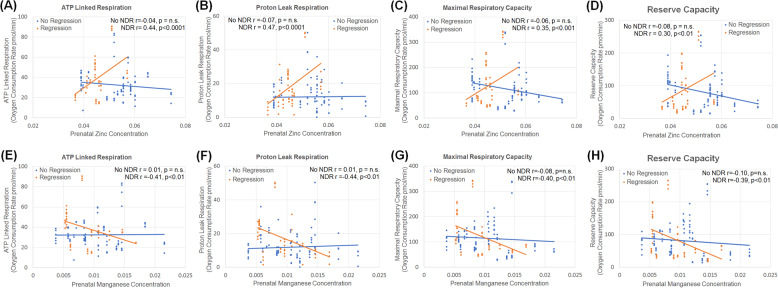
Relationship between prenatal metals and mitochondrial respiration. Relationship between the concentrations of Zinc (**a**–**d**) and Manganese (**e**–**h**) deposited in deciduous teeth prenatally and measure of mitochondrial respiration during childhood in children diagnosed with autism spectrum disorder. For all measures of mitochondrial respiration, the relationship to prenatal exposure to Zinc and Manganese was dependent on whether the child experienced neurodevelopmental regression (NDR).

Two metals, Zn and Mn, demonstrated significant relationships for three or more respiratory parameters with these relationships dependent on whether the child had a history of NDR (See Supplementary Table S3). Interestingly, when controlling for Zn and Mn concentration, all measures of mitochondrial respiration were found to be significantly higher in the children with NDR as compare with those without NDR, further demonstrating that differences in these metals significantly affect mitochondrial respiration (See Supplementary Tables S3 and S4). As seen in Fig. 2a–d, greater Zn deposited in deciduous teeth prenatally was associated with higher mitochondrial respiration for those individuals with a history of NDR but there was no significant relationship between Zn and mitochondrial respiration for those without a NDR history. As seen in Fig. 2e–h, greater Mn deposited in deciduous teeth prenatally was associated with lower mitochondrial respiration for those individuals with a history of NDR but there was no significant relationship between Mn and mitochondrial respiration for those without a history of NDR.

### Mitochondrial respiration and postnatal metal exposure

Two metals (Ni, Sr) demonstrated significant (*p* < 0.01) relationships for three or more respiratory parameters, with these relationships dependent on whether the child had a history of NDR. However, the scatterplots demonstrated that these relationships were driven by one child with particularly high concentrations of both metals. When this participant was removed, the analysis was no longer significant according to our criteria although a trend remained (data not shown).

### Glycolysis and prenatal metal exposure

**Fig. 3:**
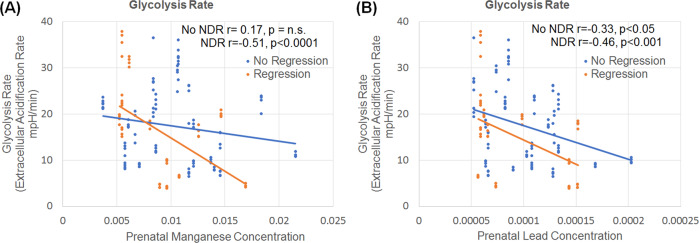
Relationship between prenatal metals and glycolysis. Relationship between Glycolytic rate and prenatal concentrations of Manganese and Lead for children with autism spectrum disorder. Prenatal concentration of (**a**) Manganese and (**b**) Lead are related to glycolysis but the relationship between metal concentrations and glycolytic rate was dependent on whether the child experienced neurodevelopmental regression (NDR).

Glycolytic rate was associated with prenatal deciduous tooth concentrations of Mn and Pb. The effect of Mn [*F*(1,113) = 6.68, *p* = 0.01] and Pb [*F*(1113) = 6.37, *p* = 0.01] on glycolytic rate was dependent on the participant’s history of NDR. The glycolytic rate decreased with an increasing concentration of Mn (Fig. 3a) and Pb (Fig. 3b) for those with a history of NDR. For those without a history of NDR, there was no significant relationship between glycolytic rate and deciduous Mn concentration while the relationship between Pb and glycolytic rate was present but weaker than those with a history of NDR.

### Glycolysis and postnatal metal exposure

**Fig. 4:**
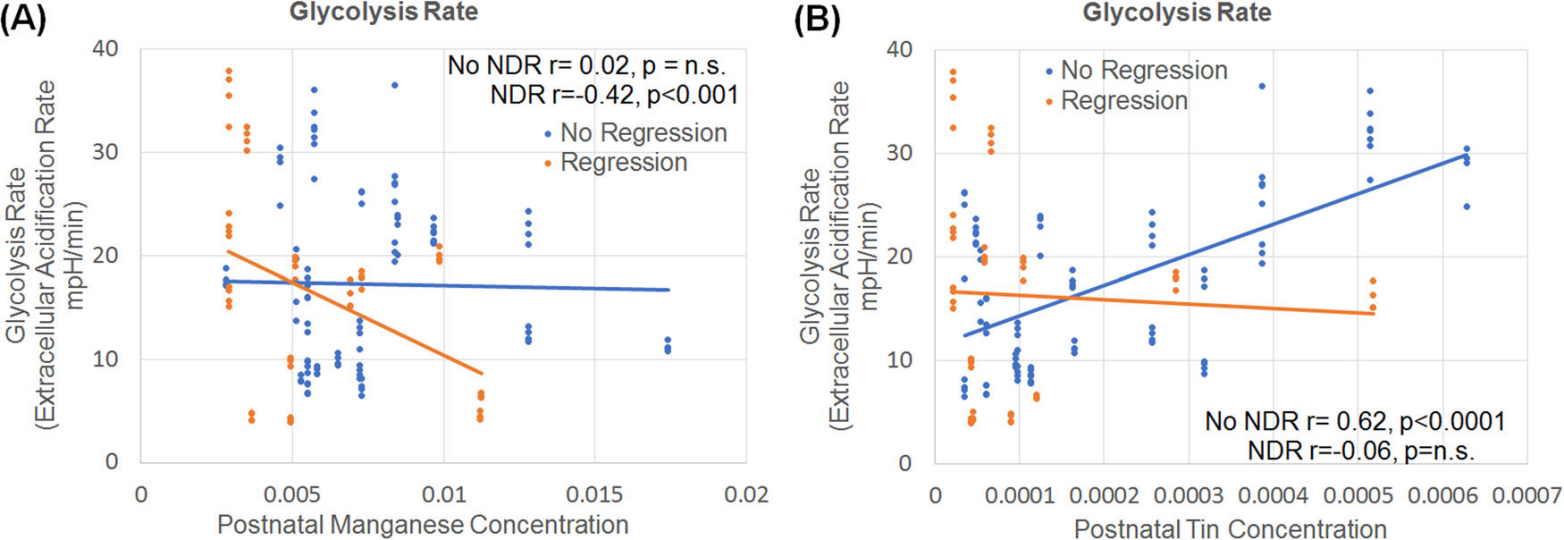
Relationship between postnatal metals and glycolysis. Relationship between Glycolytic rate and early postnatal (first 9 months of life) concentrations of (**a**) Manganese and (**b**) Tin for children with autism spectrum disorder. The relationship between metal concentrations and glycolytic rate was dependent on whether the child experienced neurodevelopmental regression (NDR).

Glycolytic rate was associated with early postnatal deciduous tooth concentrations of Mn and Sn. The effect of Mn [*F*(1113) = 9.96, *p* < 0.01] and Sn [*F*(1113) = 10.68, *p* < 0.01] on glycolytic rate was dependent on the participant’s history of NDR. The glycolytic rate decreased with an increasing concentration of Mn (Fig. 4a) for those with a history of NDR but was not associated with the glycolytic rate for those without a history of NDR. In contrast, the glycolytic rate increased with an increasing concentration of Sn (Fig. 4b) for those without a history of NDR but was not associated with the glycolytic rate for those with a history of NDR.

### Behavioral correlates of metals

**Fig. 5:**
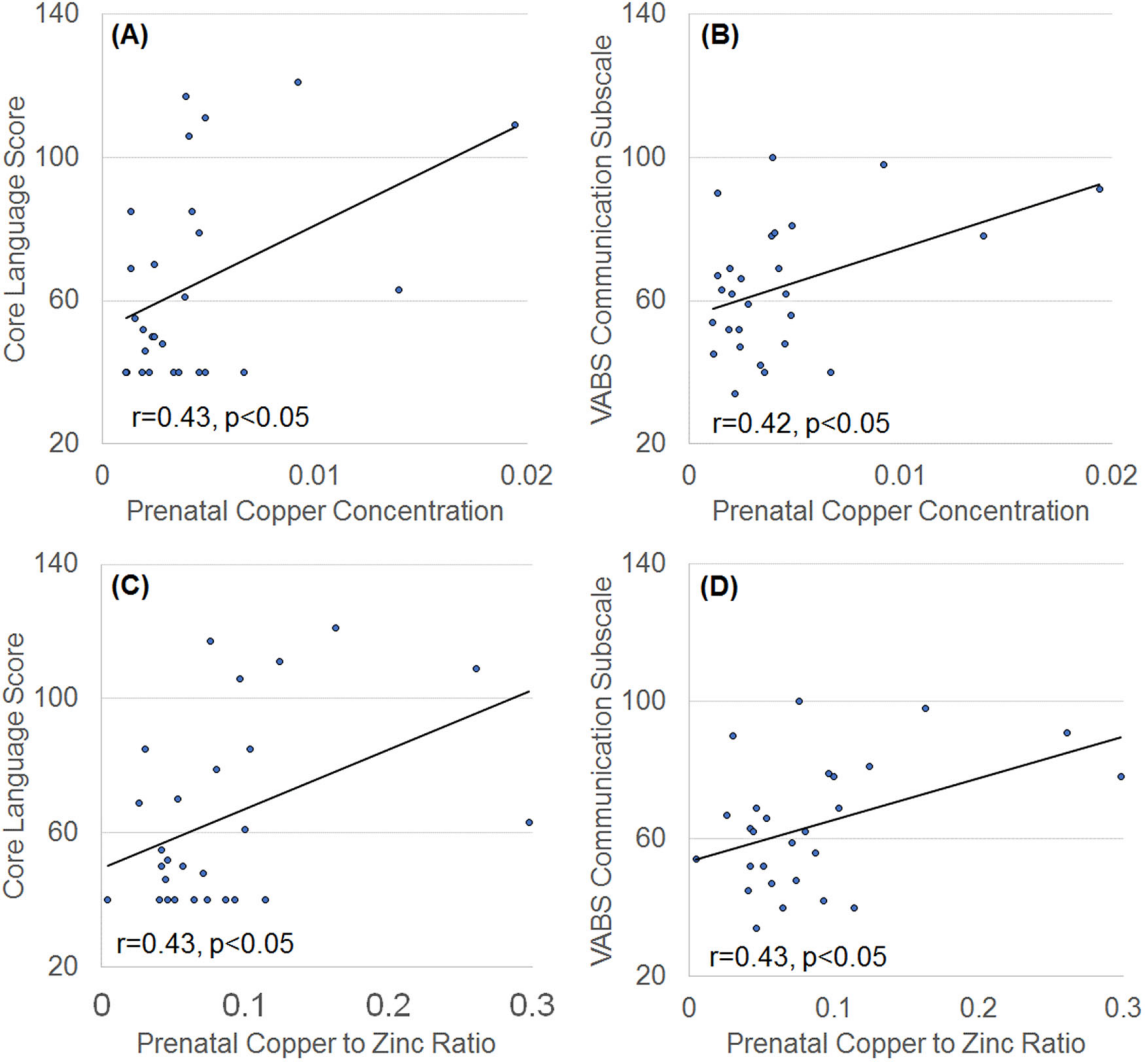
Relationship between prenatal metals and neurodevelopment. Both prenatal (**a**, **b**) copper concentration and (**c**, **d**) copper to zinc ratios correlate with (**a**, **c**) language and (**b**, **d**) communication development during childhood in children with autism spectrum disorder. VABS = Vineland Adaptive Behavior Scale.

Core language and VABS Communication subscale scores increased as prenatal Cu concentration [*F*(123) = 4.65, *p* < 0.05 and *F*(123) = 4.91, *p* < 0.05] and Cu-to-Zn ratio [*F*(123) = 6.31, *p* < 0.05 and *F*(123) = 5.76, *p* < 0.05] increased (Fig. 5).

## Discussion

Exposure to nutrient (e.g., Zn) and toxic (e.g., Pb) metals are known to be associated with ASD yet the biological mechanism by which these metals influence neurodevelopment is not well understood in humans. As both nutritional and toxic metal exposure are known to disrupt bioenergetics and bioenergetics is disrupted in ASD, it was hypothesized that prenatal and postnatal exposure to metals was associated with long-term physiological changes in mitochondrial and glycolytic metabolism. One major strength of this study is that the exact prenatal and postnatal exposure to metals were measured by examining deciduous teeth on an individual participant basis.

We examined the prenatal and early postnatal concentrations of Pb (lead), Ni (nickel), Cr (chromium), Mg (magnesium), Mn (manganese), Cu (copper), Zn (zinc), Sr (strontium), Sn (tin), and Ba (barium) by analyzing deciduous teeth. First, concentrations of metals in children with ASD were compared with TD control children. Most notable was significantly lower concentrations of Cu in individuals with ASD as compared with control children in both the prenatal and early post-natal periods. Second, the relationship between metals and four measures of mitochondrial respiration were examined in children with ASD. Both prenatal concentrations of Mn and Zn were found to be significantly related to all measures of mitochondrial respiration, but only for children with a history of NDR.

The relationship between metals and glycolytic metabolism was also investigated. Both prenatal and early post-natal Mn concentration was found to be related to glycolytic rate for those children with a history of NDR. For children with a history of NDR, prenatal Pb was also found to be associated with glycolytic rate while for children without a history of NDR postnatal Sn was associated with glycolytic rate. Lastly, the relationship between metals and neurodevelopment and behavior were investigated. Prenatal Cu and the Cu/Zn ratio, which were both abnormal prenatally in those with ASD, were found to be related to language and communication. The Pearson *r* correlation coefficients used to describe and confirm these relationships suggest that there is a medium effect size to these relationships. These results are discussed in separate sections below.

### The importance of copper and zinc metabolism

Zn deficiency has been associated with ASD both prenatally and postnatally. Two animal models of ASD involved abnormalities in Zn metabolism and demonstrate abnormalities in brain development. The prenatal Zn-deficient mouse model of ASD demonstrates enlargement of the thalamus^43^ and striatum^44^, and an absence of lateralization of dopamine receptor 1 in the striatum^44^. Male Zn transporter 3 null mice demonstrate ASD-type behaviors and increased cortical volume and neurite density^45^. Using deciduous teeth, a previous twin study demonstrated that prenatal Zn deficiency was related to ASD^11^. Several studies have demonstrated that children with ASD have lower Zn blood concentrations^46–49^ and have linked postnatal Zn concentrations during childhood to ASD severity. Indeed, one study found that hair Zn concentrations are inversely related to ASD symptom severity^50^ and another study found that Zn blood levels were positively correlated with neurodevelopment^47^.

Perhaps more compelling, but preliminary, are studies on the potential therapeutic use of Zn to treat ASD behaviors. Zn supplementation improved ASD symptom severity and serum concentrations of metallothionein-1 and Cu in an uncontrolled cohort of children with ASD^51^. Despite only one patient study, there are several studies on the use of Zn therapeutically in animal models of ASD. Prenatal Zn treatment protects the prenatal valproic acid^52^ and lipopolysaccharide^53^ exposure mouse models of ASD. Zn supplementation also prevents repetitive and anxiety behaviors in the Phelan-McDermid Syndrome mouse model of ASD^54^.

In this study we found that prenatal Zn concentrations modulates mitochondrial function in participants with a history of NDR. Recent research has identified several Zn containing low-molecular mass complexes in mitochondria^55^. The major role of Zn with respect to mitochondrial function is also related to its function as an antioxidant through several mechanisms: the Zn-thiolate moiety of metallothioneins scavenge hydroxyl radicals, Zn competes with other potentially toxic metals to prevent their detrimental biological effects and, most prominently, Zn is a cofactor for superoxide dismutase (SOD), along with Cu, to protect the cell from oxygen radicals^56^. Alternatively, high Zn concentrations can inhibit mitochondrial citric acid cycle and ETC enzymes^56^.

Abnormalities in Zn and Cu metabolism are associated with ASD with the Zn-to-Cu ratio being suggested as a biomarker of ASD^57^. However, unlike many post-natal studies which suggest that Cu is elevated and the Zn-to-Cu ratio is low in children with ASD^57^, this study found that prenatal Cu was low and Cu-to-Zn ratio was low (corresponding to a high Zn-to-Cu ratio). A recent twin study examining deciduous teeth suggested that disrupted dynamics of prenatal Zn-Cu cycles could differentiate ASD and control cases with a 90% accuracy^12^. Beyond the effect of Cu associated with Zn for SOD function, Cu is essential for the mitochondrial ETC complex IV, cytochrome oxidase^58^. In fact, cells contain specific machinery for transporting Cu into the intermembrane space of the mitochondria^58^. Alternatively, excess Cu can result in toxicity, especially in the brain^59^. Given the low levels of Cu found in the ASD participants in this study, toxicity is likely not an issue. In fact, low Cu concentrations may have limited the ability of the mitochondria to produce ATP, resulting in a background of mitochondrial dysfunction in the children studied. In addition, low Cu relative to Zn concentration most likely resulted in significant disruption of the function of important Cu-Zn enzymes.

### Manganese, an important nutrient metal with potentially toxic effects

Studies have suggested that Mn exposure is associated with neurocognitive dysfunction and psychosis^14^. Studies have associated higher Mn blood concentration during childhood with more severe ASD symptoms^60^, lower cognitive function^50^, and inflammatory markers^61^. However, using deciduous teeth, Mn concentrations have been shown to be lower in children with the ASD during the prenatal and early postnatal periods in a twin study with higher Mn concentrations correlating with less severe ASD symptoms^11^. Mn is essential for mitochondrial function due to its role in the mitochondrial specific SOD enzyme^62^, but neurons and astrocyte mitochondria are particularly sensitive to excess Mn which can lead to mitochondrial dysfunction through neuroinflammation and impaired mitochondrial repair^63^. The data from this study suggest that a higher Mn concentration is associated with reduced mitochondrial respiration and glycolysis in ASD participants with a history of NDR. Unlike the previous study using deciduous teeth, our participants with ASD did not appear to be Mn deficient. Given the narrow therapeutic window of nutritional Mn, the baseline Mn level of the participants may be an important factor associated with the modulatory effect of Mn on ASD severity and physiology.

### Lead and Tin, two metals associated with changes in glucose metabolism

Sources of Pb exposure are ubiquitous in the environment, from old mine tailings, the legacy of leaded gasoline, paint deposits to soils and air pollution. Regardless of the source of Pb, a previous twin study suggests that environmental Pb deposited in deciduous teeth during fetal life is higher in individuals with ASD^11^, and the current study suggests that it can result in long-term changes that decrease glucose metabolism in individuals with ASD and NDR. This is consistent with recent studies demonstrating that Pb exposure promotes diabetes^64^ and abnormal brain glucose metabolism^65^ in rodents, and may be related to the development of gestational diabetes in humans^66^. This study also found that early life Sn exposure was related to long-term changes in glucose metabolism in children with ASD without a history of NDR. Higher Sn concentrations have been associated with diabetes in adults^67^.

### Mitochondria dysfunction and neurodevelopmental regression

Several studies suggest that NDR is associated with mitochondrial dysfunction^24^, especially in children with ASD^16,23^. For example, Shoffner et al.^23^ showed that the majority of the children with ASD who were diagnosed with mitochondrial disease had a sudden rapid NDR, resulting in the development of ASD symptoms following an inflammatory trigger and fever. In addition, a meta-analysis of children with ASD also diagnosed with mitochondrial disease demonstrates a significantly higher rate of NDR^16^. We have developed a model of mitochondrial dysfunction in lymphoblastoid cell lines derived from patients with ASD where the mitochondria are more sensitive to reactive oxygen species as manifest by a significant loss of Reserve Capacity as reactive oxygen species is increased in vitro^20,68–74^. This subset of lymphoblastoid cell lines also respond differently to environmental agents associated with ASD including trichloroacetaldehyde hydrate^75^ and the microbiome associated short-chain fatty-acids propionate^76^ and butyrate^77^. Thus, it is likely that many children with ASD and NDR have underlying mitochondrial dysfunction which makes their mitochondria sensitive to both supportive (nutritional) and detrimental (toxic) environmental influences.

### Limitations

There are several limitations to this study, primarily the small sample size and the retrospective nature of the developmental history including the history of NDR. The small sample size limits the power of the statistical analyses. Thus, we cannot rule out relationships that are not found to be statistically significant. Power analysis suggests linear regression model with two ASD groups (NDR vs. no NDR) assuming a medium effect size, an alpha of 0.01 and a power of 80% requires at least 191 measurements. Our history questionnaire and follow-up clarification questions obtain a very detailed account of the NDR including developmental level prior to the NDR, the skills lost and the acuity of the event. Further research will be needed to identify the significance of these physiological changes on long-term disease.

The relationship between metal and neurodevelopment and behavior were investigated. Cu and the Cu/Zn ratio was found to be related to language and communication. These metals were found to be abnormal prenatally in those with ASD but were not related to changes in mitochondrial respiration. Metals associated with changes in mitochondrial respiration were not strongly associated with behavior in this study but previous studies have documented a connection between mitochondrial function and neurodevelopment and behavior in children with ASD^16,29^ suggesting that the mitochondrial could be the mediator between any effect of some metals on neurodevelopment and behavior in children with ASD. The number of subjects in this study is too small to test such a hypothesis, but larger studies may be able to answer this question in the future.

## Conclusion

Although preliminary, this study demonstrates that early-life exposure to nutritional and toxic metals can alter long-term bioenergetics, possibly resulting in disease or increasing vulnerability to disease triggered by later life events. This study found that exposure to metals during gestation was associated with variation in bioenergetic metabolism during childhood in children with ASD and NDR. The sources of the exposure have not been examined in this study. The prenatal concentrations of metals measured in deciduous teeth that were found to be significantly related to ASD, long-term change in mitochondrial respiration and/or language development (i.e., Zn, Cu, Mn) are mostly derived from the maternal diet, suggesting that monitoring of such metals during the prenatal period may be important to ensuring optimal mitochondrial function and neurodevelopment in the fetus.

Aside from dietary sources, air pollution, which has been linked to ASD, contain many metals including naturally sourced crustal elements: Ca, Mg, Na, K, Al, Si, Fe, Ti, Mn and anthropogenically sourced metals: V, Cr, Ni, Zn, Cd, Pb, Cu^78,79^. Abnormalities in mitochondrial metabolism can be long-lasting and are implicated in a wide variety of diseases, which are suspected to have an environmental component, including psychiatric, neurodevelopmental, and neurodegenerative disorders, as well as systemic inflammation, cardiac disease, cancer, and diabetes. This study suggests that it is important to monitor the effects of sources of metal exposure on the growing fetus in order to optimize health and development.

## Supplementary information

Supplementary Information
