# Supplementary material for: Early life metal exposure dysregulates cellular bioenergetics in children with regressive autism spectrum disorder
Source: Transl Psychiatry. 2020 Jul 7;10:223. doi: 10.1038/s41398-020-00905-3 (PMC7341836; doi:10.1038/s41398-020-00905-3)
Supplement: Supplementary file 1 — Supplementary Information [file 41398_2020_905_MOESM1_ESM.docx]

**Supplemental Material**

| Supplementary Table 1. Participant Characteristics | |  |  |
| --- | --- | --- | --- |
| Variable | Typically Developing (n=7) | ASD without  Regression (n=14) | ASD with  Regression (n=13) |
| Age, mean (SD), years months | 8 y 6 m (2 y 6 m) | 10y 9m (3y 10m) | 10y 11m (3y 3m) |
| Males, N (%) | 2 (29%) | 7 (54%) | 6 (43%) |
| ASD Diagnostic Documentation, N (%) |  |  |  |
| Autism Diagnostic Observation Schedule (ADOS) |  | 3 (14%) | 5 (38%) |
| Autism Diagnostic Interview-Revised (ADI-R) |  | 4 (29%) | 4 (31%) |
| Gold Standard Diagnosis  (ADI-R or ADOS) |  | 7 (50%) | 8 (62%) |
| Diagnosis by physician, psychologist, and speech therapist (Arkansas State Standard) |  | 6 (42%) | 3 (23%) |
| DSM diagnosis by physician with standardized, validated questionnaires & diagnosis confirmation by the Principal Investigator |  | 7 (50%) | 9 (69%) |
| Regression, N (%) |  |  | 13 (100%) |
| Single Regression |  |  | 10 (77%) |
| Age at 1^st^ Regression |  |  | 27m (18m) |
| Lost of Language Skills |  |  | 10 (77%) |
| Lost of Social Skills |  |  | 8 (62%) |
| Lost of Gross Motor Skills |  |  | 3 (23%) |
| Lost of Fine Motor Skills |  |  | 3 (23%) |
| Associated Fever |  |  | 0 (0%) |
| Associated Illness |  |  | 4 (31%) |
| Associated Seizure |  |  | 3 (23%) |
| Core Language (Scaled Score) | 94 (5) | 64 (31) | 65 (24) |
| Vineland Adaptive Behavior Scale (Scaled Score) |  |  |  |
| - Communication Subscale | 113 (8) | 63 (20) | 65 (17) |
| - Daily Living Skills Subscale | 111 (13) | 63 (21) | 66 (14) |
| - Social Subscale | 119 (8) | 62 (20) | 61 (15) |
| - Motor Subscale | 107 (12) | 75 (22) | 79 (17) |
| - Adaptive Behavioral Composite | 115 (7) | 62 (19) | 63 (15) |
| Social Responsiveness Scale (T-Score) |  |  |  |
| - Awareness | 52 (10) | 77 (15) | 76 (8) |
| - Cognitive | 53 (16) | 82 (14) | 82 (9) |
| - Communication | 52 (14) | 81 (15) | 81 (6) |
| - Motivation | 56 (16) | 74 (14) | 74 (8) |
| - Mannerisms | 53 (18) | 83 (13) | 87 (5) |
| - Total | 54 (16) | 83 (14) | 85 (5) |
| Aberrant Behavior Checklist (Raw Score) |  |  |  |
| - Irritability | 5 (7) | 18 (11) | 16 (7) |
| - Lethargy / Social Withdrawal | 3 (3) | 12 (7) | 12 (6) |
| - Stereotyped Movements | 1 (3) | 7 (7) | 6 (5) |
| - Hyperactivity | 10 (12) | 22 (13) | 17 (9) |
| - Inappropriate Speech | 1 (1) | 3 (3) | 5 (4) |
| Comorbid Conditions (Parent Report), N (%) |  |  |  |
| Neurologic | 2 (29%) | 13 (93%) | 10 (77%) |
| Allergic | 5 (71%) | 5 (36%) | 3 (23%) |
| Psychiatric | 1 (14%) | 10 (71%) | 6 (46%) |
| Gastrointestinal | 3 (43%) | 10 (71%) | 8 (62%) |
| Immune | 1 (14%) | 12 (86%) | 10 (77%) |
| Growth | 2 (28%) | 8 (58%) | 3 (23%) |
| Endocrine | 0 (0%) | 2 (14%) | 1 (8%) |
| Cardiovascular | 0 (0%) | 1 (7%) | 2 (15%) |
| Comorbid Conditions (Medical Records), N (%) |  |  |  |
| Food Allergies/Intolerances | 2 (29%) | 11 (79%) | 10 (77%) |
| Epilepsy | 0 (0%) | 3 (21%) | 6 (46%) |
| Chronic Constipation | 0 (0%) | 8 (57%) | 8 (62%) |
| Fatigue/Exercise Intolerance | 1 (14%) | 7 (50%) | 6 (46%) |
| Recurrent Infections (AAAAI Criteria) | 1 (14%) | 6 (43%) | 4 (31%) |
| Gross Motor Delay | 0 (0%) | 7 (50%) | 9 (69%) |
| Hypogammaglobinemia | 0 (0%) | 1 (7%) | 1 (8%) |
| Failure to Thrive | 0 (0%) | 0 (0%) | 4 (31%) |
| Genetic Syndrome | 0 (0%) | 3 (21%) | 1 (8%) |
| Treatments, N (%) |  |  |  |
| Gastrointestinal Medications | 3 (42%) | 5 (36%) | 10 (77%)* |
| Mineral Supplements | 0 (0%) | 2 (14%) | 6 (46%) |
| Melatonin | 0 (0%) | 6 (43%) | 3 (23%) |
| Allergy/Asthma Medications | 1 (14%) | 5 (36%) | 3 (23%) |
| Antiepileptic Medications | 0 (0%) | 3 (21%) | 6 (46%) |
| Antimicrobial Medications | 0 (0%) | 3 (21%) | 4 (31%) |
| Antipsychotic Medications | 0 (0%) | 3 (21%) | 0 (0%) |
| Immunomodulatory Medications | 0 (0%) | 0 (0%) | 1 (8%) |
| Other Psychotropic Medications | 0 (0%) | 2 (14%) | 2 (15%) |
| Hormone Supplementation | 0 (0%) | 1 (7%) | 1 (8%) |
| Stimulant | 0 (0%) | 2 (14%) | 2 (15%) |
| Thyroid Supplementation | 0 (0%) | 2 (14%) | 0 (0%) |
| Alpha-adrenergic agonists | 0 (0%) | 1 (7%) | 0 (0%) |
| Selective Serotonin Reuptake Inhibitors | 0 (0%) | 2 (14%) | 2 (15%) |
| Beta Blocker | 0 (0%) | 1 (7%) | 1 (8%) |
| Dietary Formula | 0 (0%) | 0 (0%) | 2 (15%) |

Medication not taken by any participant include Anticholinergic, Diuretic, and Muscle Relaxers.

*p=0.05 between ASD groups by Fisher Exact test.

| Supplementary Table S2. Metal concentrations [Mean (SD)] in deciduous teeth during the prenatal and early postnatal period in typically developing controls and children with ASD, some with neurodevelopmental regression (NDR+) and some without neurodevelopmental regression (NDR-). Significant differences are bolded and underlined. | | | | | | | | | | | |
| --- | --- | --- | --- | --- | --- | --- | --- | --- | --- | --- | --- |
|  | Mg | Cr | Mn | Ni | Cu | Zn | Cu/Zn | Sr | Sn | Ba | Pb |
| Prenatal |  |  |  |  |  |  |  |  |  |  |  |
| ASD (n=14)  NDR- | 1.051  (0.143) | 0.00285  (0.00047) | 0.0110  (0.0064) | 0.00010  (0.00014) | 0.0045  (0.0046) | 0.0617  (0.0562) | 0.0868  (0.0600) | 0.624  (0.840) | 0.00014  (0.00010) | 0.0164  (0.0070) | 0.00011  (0.00005) |
| ASD (n=13)  NDR+ | 1.062  (0.163) | 0.00280  (0.00036) | 0.0100  (0.0039) | 0.00008  (0.00004) | 0.0040  (0.0037) | 0.0474  (0.0072) | 0.0825  (0.0742) | 0.441  (0.170) | 0.00010  (0.00005) | 0.0165  (0.0067) | 0.00016  (0.00017) |
| ASD (n=27)  Overall | 1.056  (0.150) | 0.00282  (0.00041) | 0.0105  (0.0052) | 0.00009  (0.00009) | 0.0043  (0.0041) | 0.0548  (0.0548) | 0.0847  (0.0659) | 0.536  (0.612) | 0.00012  (0.00008) | 0.0164  (0.0067) | 0.00013  (0.00012) |
| TD (n=7)  Controls | 0.943  (0.106) | 0.00258)  (0.00385) | 0.0122  (0.0044) | **0.00029**  (0.00023) | **0.0150**  (0.0086) | 0.0580  (0.0118) | **0.2557**  (0.1179) | 0.487  (0.128) | 0.00022  (0.00018) | 0.022  (0.008) | 0.000115  (0.00004) |
| Postnatal |  |  |  |  |  |  |  |  |  |  |  |
| ASD (n=14)  NDR- | 1.070  (0.210) | 0.00268  (0.00062) | 0.0081  (0.0034) | 0.00010  (0.00014) | 0.00340  (0.0034) | 0.0525  (0.0357) | 0.0804  (0.0579) | 0.647  (0.687) | 0.00013  (0.00011) | 0.0200  (0.0082) | 0.00023  (0.00042) |
| ASD (n=13)  NDR+ | 1.150  (0.223) | 0.00254  (0.00084) | 0.0070  (0.0034) | 0.00010  (0.00014) | 0.0039  (0.0034) | 0.0477  (0.0119) | 0.0743  (0.0457) | 0.571  (0.273) | 0.00014  (0.00015) | 0.0219  (0.0091) | 0.00021  (0.00017) |
| ASD (n=27)  Overall | 1.109  (0.216) | 0.00261  (0.00072) | 0.0076  (0.0034) | 0.00010  (0.00014) | 0.0039  (0.0034) | 0.0502  (0.0266) | 0.0775  (0.0515) | 0.610  (0.522) | 0.00014  (0.00013) | 0.0209  (0.0085) | 0.00022  (0.00032) |
| TD (n=7)  Controls | 0.981  (0.116) | 0.00202  (0.00065) | 0.0063  (0.0024) | 0.00021  (0.00021) | **0.0136**  (0.0130) | 0.0599  (0.0103) | 0.2298  (0.2277) | 0.554  (0.167) | 0.00026  (0.00022) | 0.027  (0.008) | 0.00017  (0.00010) |

TD children demonstrated significantly higher Nickel (Ni) [F(1,33)=11.56,p<0.01], Cu [F(1,33)=23.48,p<0.001] and Cu-to-Zn Ratio [F(1,32)=9.21,p<0.01] as compared to ASD children during the prenatal period and higher Cu [F(1,33)=12.59, p=0.001] as compared to ASD children during the early postnatal period. Zn [F(1,33)=5.84, p<0.05] and Sn [F(1,33)=4.88, p<0.05] were also higher in TD controls during the prenatal although the difference did not reach our stringent (p<=0.01) criteria.

| Supplementary Table S3. Regression analysis statistics for metals with statistical significance (p<=0.01) for 3 or more respiratory parameters. | | | | |
| --- | --- | --- | --- | --- |
|  | Mean Difference | | Interaction | |
|  | Zn | Mn | Zn | Mn |
| Adenosine-triphosphate Linked Respiration (ALR) | F(1,113)=12.31, p=0.001 | F(1,113)=7.79, p<0.01 | F(1,113)=14.49, p<0.0001 | F(1,113)=5.34, p<0.05 |
| Proton Leak Respiration (PLR) | F(1,113)=11.12, p=0.001 | F(1,113)=14.25, p<0.0001 | F(1,113)=13.31, p<0.0001 | F(1,113)=9.19, p<0.01 |
| Maximal Respiratory Capacity (MRC) | F(1,113)=8.16, p<0.01 | F(1,113)=7.54, p<0.01 | F(1,113)=8.57, p<0.01 | F(1,113)=8.07, p<0.01 |
| Reserve Capacity (RC) | F(1,113)=6.31, p=0.01 | F(1,113)=6.07, p=0.01 | F(1,113)=6.30, p=0.01 | F(1,113)=7.47, p<0.01 |

| Supplementary Table S4. Mean (SD) of respiratory measures for children with ASD with and without neurodevelopmental regression | | |
| --- | --- | --- |
|  | ASD without Neurodevelopment Regression | ASD with Neurodevelopment Regression |
| Adenosine-triphosphate Linked Respiration (ALR) | 33.0 (14.6) | 38.8 (19.6 |
| Proton Leak Respiration (PLR) | 12.5 (10.9) | 18.0 (12.0) |
| Maximal Respiratory Capacity (MRC) | 120.5 (66.6) | 127.0 (85.5) |
| Reserve Capacity (RC) | 86.8 (54.7) | 86.8 (70.2) |
